# Understanding the preference of online health information seeking among college students using the best-worst scaling method

**DOI:** 10.3389/fpubh.2025.1670106

**Published:** 2025-11-05

**Authors:** Dan Wang, Wang Jiang, Haihong Chen, Manli Chen, Guangwen Gong, Yansun Sun, Yajing Wu, Xuemei Wang, Xiping Li

**Affiliations:** ^1^School of Management, Hubei University of Chinese Medicine, Wuhan, China; ^2^School of Health Policy and Management, Nanjing Medical University, Nanjing, China; ^3^Department of Geriatric Medicine, Peking University Shenzhen Hospital, Shenzhen, China; ^4^Nanjing Drum Tower Hospital, The Affiliated Hospital of Nanjing University Medical School, Nanjing, China

**Keywords:** online health information seeking, preference, college students, best-worst scaling, accuracy, credibility

## Abstract

**Background:**

With the overwhelming availability of online health information and high prevalence of health misinformation, it is vital to understand the status and key influencing factors of its use among individuals. This study aims to explore the online health information-seeking behavior and preference of the influencing factors among college students.

**Methods:**

We used the best-worst scaling approach to determine college students’ preferences for factors influencing online health information-seeking behavior. A total of 11 attributes of online health information seeking were confirmed by literature review and focus group, and a balanced incomplete block design was used to create 11 tasks for the BWS survey. An online survey was conducted from March 2023 to May 2023 using the BWS survey questionnaire.

**Results:**

Both the BWS score and mixed logit model results indicate that “verified by professional institutions or health professionals”(mean BW=1.938; coefficient = 3.096), “information source from trustworthy and authoritative website”(mean BW = 1.921; coefficient = 3.015), “privacy and security guaranteed”(mean BW = 1.234; coefficient = 2.637), and “consistency of information” (mean BW = 0.803; coefficient = 2.313) were the most important factors and were valued more positively than negatively by respondents. The results showed the covariate of medical education had positive effects of 0.410 and 0.279 on the preference of “writing and language” and “professional interface design,” while medical education background had negative effects of −0.307 on the preference of “disclosure of author information.”

**Conclusion:**

We recommend that concerned authorities consider interventions targeting the accuracy, credibility, privacy, and consistency of online health information management for college students.

## 1 Introduction

With the improvement of digital technology and the advantages of convenience, immediacy, and interactivity of the Internet, an increasing trend of online health information seeking has been observed among the public (1). Online health information seeking serves multiple purposes for consumers, such as understanding disease symptoms, finding treatment choices, and preparing for patient–doctor communication and lifestyle modifications (2). The positive association of online information seeking and better health outcomes after obtaining adequate information on health conditions has been reported by a number of researchers previously (3–5). In addition, online information seeking was also reported to enhance consumers’ communication ability and improve physician–patient relationships and patients’ satisfaction (6, 7).

Online health information seeking has become a prevalent way for consumers to obtain health information. One cross-sectional survey in 2014 showed that approximately 60% of young European adults searched online for health information (8). One national survey conducted in the United States showed that 69.8% ~ 81.5% had searched for health or medical information online across years from 2008 to 2017 (9). The proportion of online health information seeking was higher among college students. College students are in high need of proficient health information. One national study of 1,277 university students in Greece showed that 90% of the respondents used the Internet to seek health information (10). One study of 1,203 samples measuring Chinese college students’ online health information behavior showed that more than 90% of the respondents had online health information-seeking behavior. College students relied heavily on online health information seeking to manage their own and others’ (family members, friends) health conditions (11). Especially, COVID-19 pandemic, accompanied by a pervasive infodemic, has profoundly reshaped health information-seeking behavior, solidifying the Internet as a primary source of real-time health guidance (12). With a high prevalence of health information-seeking behavior and characterized by high digital immersion and acute vulnerability to misinformation, college students have emerged as a population of particular concern and research focus (13, 14).

While the accessibility and convenience of online health information offer significant advantages, it also exposes individuals to a deluge of potentially misleading or false content. The pervasive nature of health misinformation poses a significant challenge for individuals in distinguishing credible sources from inaccurate claims. Health misinformation through the Internet has become a major public health concern (15). With higher exposure of information flow over the Internet, young adults were more likely to be affected by health misinformation or even shared health misinformation online, especially under the rapid development of social media platforms, such as TikTok and WeChat (16). According to one recent review, the high prevalence of health misinformation concerning the topics of vaccines and drugs or smoking reached 32 and 22%, respectively, by means of social media (17).

This overwhelming availability of online health information and the high prevalence of health misinformation highlights the importance of understanding the status and key influencing factors of its use among individuals (18). College students are in the critical developmental stage to make informed healthcare decisions when they are suffering from different health symptoms, illness, or injuries (11). With a high level of competency in Internet use with digital technologies, they are more likely to search for and be affected by online health information (19, 20). Therefore, it is vital to understand college students’ online health information-seeking behavior and what they value most their online health information-seeking behavior.

Despite the widespread use of online health information, there is a lack of clear understanding regarding which specific factors college students prioritize when evaluating the credibility and usefulness of this information, especially in light of pervasive health misinformation. While previous studies have identified a range of influencing factors, they often simply catalogue these elements without delving into their relative weight. As a result, a large number of items concerning the content of the information and its accuracy, comprehensiveness, currency, information source and its credibility, and the design of online information were explored and identified as affecting consumers’ online health information based on previous studies (3, 21, 22). Given such a large list of items, ranking the entire list would have been difficult, and rating items one at a time would not provide insights into their relative importance. Compared with traditional rating scales, using the best-worst scaling (BWS) method is considered superior, as it avoids response biases and allows for more reliably capturing the participants’ preferences and relative importance of factors (23–27). Traditional Likert scales are prone to response biases, such as central tendency bias, where participants may rate all items similarly high, making it difficult to discern genuine priorities. In contrast, BWS addresses this through repeated choice tasks where participants select the most and least important factors within experimentally designed choice sets. This forced-choice design is particularly suited to the objective of this study, understanding the complex decision-making landscape of college students’ health information seeking. By simulating real-world scenarios where individuals must prioritize competing factors, BWS more reliably generates a clear hierarchy of the relative importance of influencing factors.

Therefore, this study aims to explore college students’ online health information-seeking behavior and identify the relative importance of factors influencing this behavior using the best-worst scaling (BWS) method.

## 2 Materials and methods

### 2.1 Generation of BWS factors

The literature review and one focus group were conducted for generating the critical factors influencing college students’ online health information-seeking behavior in the BWS survey.

### 2.2 Literature review

The research exploring barriers, facilitators or other factors related to college students’ online health information behavior was searched and screened. Two researchers (DW and JW) carried out the literature review using Medline, Embase, and Web of Science with searching terms of “health information seeking,” HISB, “health information seeking behavior,” “Internet,” “online,” “website,” “attribute,” “factor,” or “element.” Inclusion criteria included: Studies that focus on the influencing factors of health information-seeking behavior, particularly those pertaining to source-related characteristics such as information accessibility and information quality, were included. The searching contents were not limited to university or college students to generate a more comprehensive and complete list of influencing factors. Studies were included if they were published in English and between 2000 and 2025. Exclusion criteria: Studies were excluded if their primary aim was to investigate the mediating or moderating mechanisms, or the causal/associative pathways, between online health information-seeking behavior and individual-level characteristics (e.g., socio-demographic factors or psychosocial factors). Studies that focused on the health information-seeking behavior of healthcare workers were excluded. Editorials, letters, commentaries, or conference abstracts were excluded. Studies not published in English were excluded. We identified 3,069 potentially eligible published articles, of which 33 met our inclusion criteria. Finally, a total of 16 influential factors were identified. The PRISMA flow diagram and the resulting 16 online health information-seeking attributes are presented in Supplementary Files (Figure S1 and Table S1).

The second stage involved one focus group to further review the screened factors for the BWS survey. By convenience sampling, 12 college students from medical colleges (6 students) and non-medical colleges (6 students) with experience of online health information-seeking behavior in the last month were recruited. The participants were asked to discuss the screened factors and rank these factors based on their experience of online health information-seeking behavior. Based on the results of the literature review and focus group, a refined list of 11 factors was confirmed for this study. The final list of the chosen factors and detailed descriptions of the factors are presented in Table 1.

**Table 1:** BWS attributes and descriptions of online health information seeking.

BWS attributes	Attribute description
Information source from trustworthy and authoritative website	The health information comes from authoritative and professional websites.
Verified by professional institutions or health professionals	The health information has been verified by professional medical institutions or health professionals, such as registered doctors.
Recommendations from other users	The health information is recommended by other users, such as high-ranking answers or personal experience in social media.
Disclosure of the site owner, website disclaimer, and contact information	The site owner, disclaimer (private or non-private sites), and contact information are disclosed on the website.
Writing and language	Simple, plain, straightforward language is used to help users understand health information.
Disclosure of author information	The listing authors and authors’ credentials are explicitly disclosed on the website.
Consistency of information	Health information from different sources is with high consistency. For example, the treatment principles are consistent from different platforms.
Currency of information	The health information is released within the last 3 years.
Privacy and security guaranteed	The platform guarantees the privacy and security of individual information.
Professional interface design	The overall appearance is professional, and with a navigation function for providing users access to further details and sources.
Without advertisement links	There are no advertisement links on the information interface.

### 2.3 Questionnaire design

**Figure 1:**
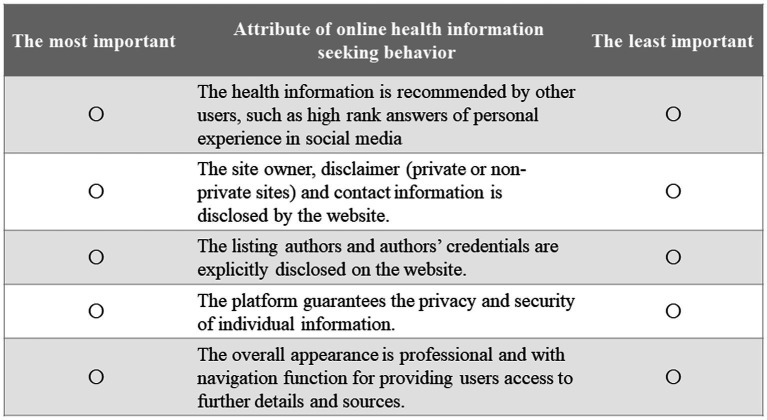
Example of online health information seeking BWS task.

In this study, BWS Case 1 was applied to explore the preferences regarding a particular list of objects, which has been widely applied in many academic fields, such as marketing, healthcare, and food research (26–28). Following the guidance of BWS Case 1, a balanced incomplete block design (BIBD) was used to create the required BWS tasks (27). In a BIBD, each attribute appears the same number of times across all BWS tasks (five in this case). In this study, the BIBD led to 11 tasks, and each task contained 5 items (attributes). Each set of five attributes was presented singly as one BWS choice task (Figure 1). Respondents were asked to choose the best and worst of 5 items (attributes) in each of the 11 BWS tasks. The remaining part of the survey consisted of two sections on online health information-seeking behavior (frequency, searching ways, contents, and purpose of online health-seeking information) and sociodemographic characteristics of the respondents. After the completion of the BWS survey questionnaire, a pilot study was conducted among a small sample of college students in Wuhan, Hubei Province, and the pilot study results showed the good readability, understandability, and feasibility of this BWS survey questionnaire among college students.

### 2.4 Data collection

The data were collected through an online survey enrolling a sample of college students from March 2023 to June 2023. College students from four colleges (two medical colleges and two non-medical colleges) from Hubei province and Jiangsu province were invited to complete the survey. Specifically, college students with public management and literature majors from non-medical schools were invited, while college students with pharmacology and health management majors were enrolled. The survey invitation links were sent to the college students by distributing the links to the University Online Class Group, who then responded to the questionnaire through the electronic links.

Before the initiation of the data collection process, participants were asked to complete the informed consent form. Participants who consented to participate were provided with a valid link to the questionnaire. The initial introduction also explicated that the questionnaire was anonymous and that all the information collected were used exclusively for the purpose of the study. According to a review of best-worst scaling surveys in the healthcare field, with a mean sample size of approximately 400, our study defined our target sample size of 600, which meets the demand of BWS surveys (29). Ultimately, a total of 733 undergraduate students were invited to complete the survey.

### 2.5 Data analysis

BWS has several analytical methods. Both the counting approach and the modeling approach based on random utility theory were applied in this study for BWS analysis. The counting approach was used to calculate best-minus-worst scores (how many times an outcome was selected as best or worst) for each of the 11 attributes. A positive B-W score implies that the times that an attribute is chosen as most important are more than those when it is selected as least important. As for the modelling approach, the respondents were asked to evaluate all the possible pairs of options (20 possible best-worst pairs) for each task and choose the pair of best and worst simultaneously (MaxDiff model). In this study, based on MaxDiff model results, mixed logit analysis was conducted for considering the preference heterogeneity among the respondents. To explore preference heterogeneity among college students, we used a mixed logit model, which accommodates this diversity by assuming that preference parameters are randomly distributed in the population; the model thus captures heterogeneity by estimating the standard deviation of these parameter distributions (30). The shares of importance were also calculated for each attribute, representing the forecasted probability for each attribute chosen as the most important. The shares of preference show the importance of one alternative option over other alternatives on a ratio scale, and the total shares sum to one across all the 11 attributes. In addition, the effects of medical education background (students receiving medical education and students not receiving medical education) on the preference of the 11 attributes were also measured for using the random logit models. Cases with missing choices were excluded from the final analysis. All analyses were performed using R version 3.3.1.

### 2.6 Ethics approval and consent to participate

The study was approved by the Ethics Committee of Tongji Medical College, Huazhong University of Science and Technology (IORG: IORG 0003571). The written informed consent was obtained from all the respondents.

## 3 Results

### 3.1 Respondent characteristics

A total of 543 respondents completed the survey with a response rate of 74.1%. Nine respondents were excluded due to missed choices, with a valid response of 534 respondents. Among 534 samples, 77.7% were female, and 52.62% were from a medical school receiving medical education. Over 90% of respondents had online health information-seeking behavior. Of them, 33.9% had online health information-seeking behavior at least one time each week. Baidu search engine (83.90%) and social media (71.54%) have been the main ways of online health information seeking. Healthy lifestyle or behavior related (66.10%) and disease symptoms, diagnosis, and treatment related (64.79%) were the main themes of online health information seeking. In addition, mental health, such as depression and anxiety, was also searched by approximately half of respondents. As for the purpose of online health information seeking, the appearance of new signs of health conditions (76.97%) and changing health-related lifestyle or behavior (61.99%) were the most frequently chosen purposes for seeking online health information (Table 2).

**Table 2:** Respondents’ socioeconomic characteristics and online health information-seeking behavior (*n* = 534).

Characteristics	*n* (%) /Mean, SD
Gender	
Male	119 (22.28)
Female	415 (77.72)
Age (mean, SD)	20.6 (1.47)
Medical education background	
Yes	281 (52.62)
No	253 (47.38)
Frequency of online health information seeking	
> = 1 time each week	181 (33.90)
1 ~ 3 times each month	209 (39.14)
1 time several months	114 (21.35)
Seldom or never	30 (5.62)
Online Platform of online health information seeking	
Baidu (The largest general search engine)	448 (83.90)
Medical interactive platform (such as good doctors)	145 (27.15)
Social media	382 (71.54)
The individual account of registered doctors	83 (15.54)
Official website of hospitals	132 (24.72)
The content of online health information seeking	
Healthy lifestyle or behavior related.	353 (66.10)
Mental health related, such as depression and anxiety	284 (53.18)
Disease symptoms, diagnosis, and treatment related	346 (64.79)
Medication use related	283(53.00)
Doctors, hospitals, and public health sectors information	161 (30.15)
The purpose of online health information seeking	
Acquire medical knowledge out of curiosity	286 (53.56)
Change health-related lifestyle or behavior	331 (61.99)
Appear new signs of health condition	411 (76.97)
Choose medical institutions or doctors	80 (14.96)
Prepare for seeing a doctor in advance	207 (38.76)
Inquire disease diagnosis, treatment, or prescription information provided by doctors	223 (41.7)

### 3.2 BWS analysis for online health information seeking

**Figure 2:**
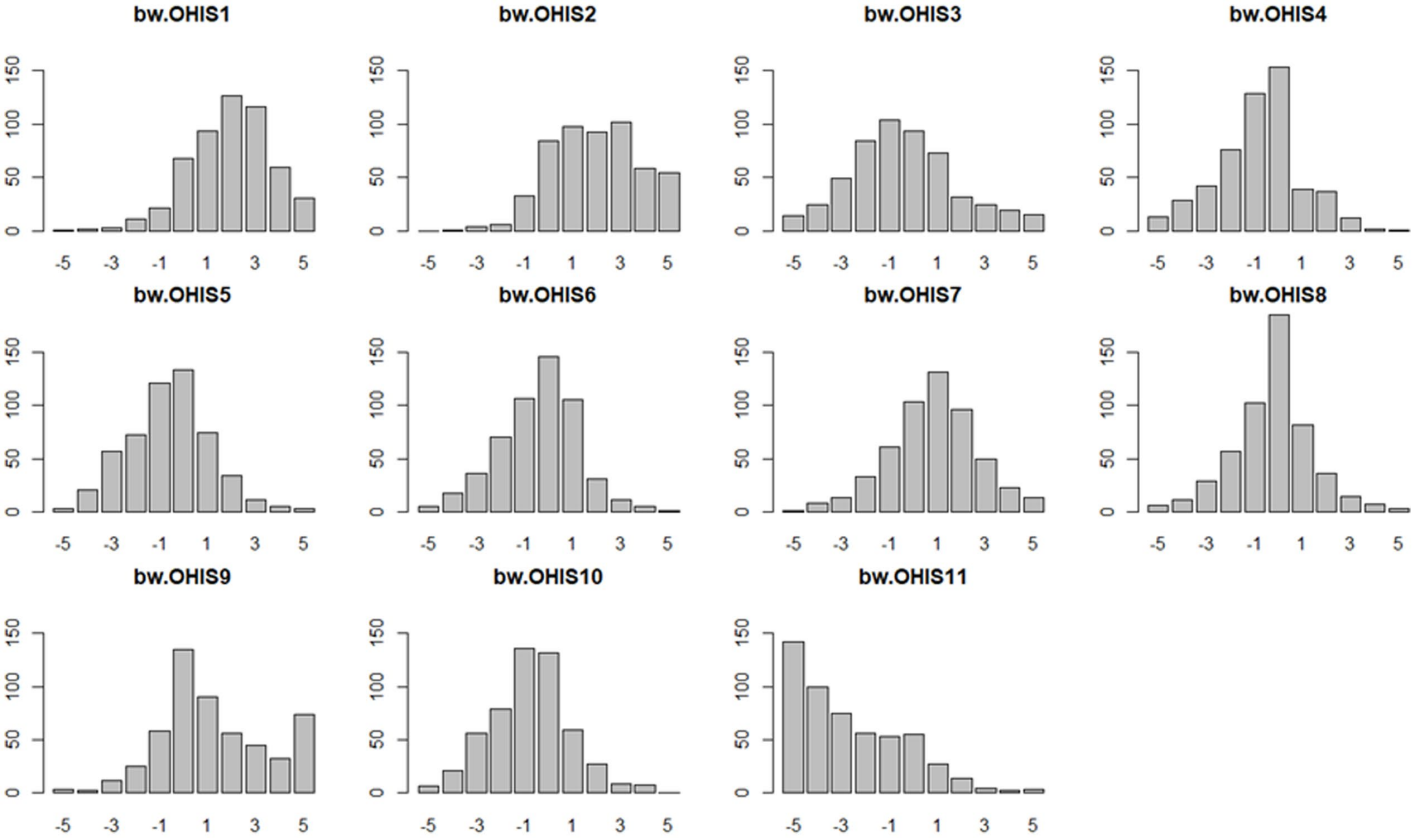
BWS scores of each attribute of online health information seeking. BWS attributes: OHIS1, Information source from trustworthy and authoritative website; OHIS2, Verified by professional institutions or health professionals; OHIS3, Recommendations from other users; OHIS4, Disclosure of site owner, website disclaimer and contact information; OHIS5, Writing and language; OHIS6, Disclosure of author information; OHIS7, Consistency of information; OHIS8, Currency of information; OHIS9, Privacy and security guaranteed; OHIS 10, Professional interface design; OHIS 11, Without advertisement links.

The best-worst scores and the standardized ratio scale of all the 11 attributes are presented in Table 3. “Verified by professional institutions or health professionals” was the most important attribute influencing online health information seeking (mean BW = 1.938). The top ranking of important attributes also included “information source from trustworthy and authoritative website” (mean BW = 1.921), “privacy and security guaranteed” (mean BW = 1.234), and “consistency of information” (mean BW = 0.803). Four out of 11 online health information-seeking attributes were valued more positively than negatively by respondents. The least important attribute factor was “without advertisement links” (mean BW = –2.618). Figure 2 presented the best-worst scores of each attribute of online health information seeking.

**Table 3:** B-W scores of online health information seeking and mixed logit model results.

BWS attributes^†^	Aggregate B-W	Mean BW	Sd of mean B-W	Sqrt B-W	Standardized ration scale	Coeff	Standard deviation	Share of preference
OHIS1	1,026	1.921	0.384	2.982	1.000	3.015**	1.003	0.223
OHIS2	1,035	1.938	0.387	2.897	0.975	3.096**	1.178	0.242
OHIS3	−226	−0.423	−0.085	0.815	0.273	1.540**	1.369	0.051
OHIS4	−426	−0.798	−0.160	0.595	0.200	1.187**	0.861	0.036
OHIS5	−325	−0.609	−0.122	0.682	0.229	1.336**	0.718	0.042
OHIS6	−226	−0.423	−0.085	0.758	0.254	1.438**	0.632	0.046
OHIS7	429	0.803	0.161	1.581	0.530	2.313**	0.983	0.110
OHIS8	−139	−0.260	−0.052	0.826	0.277	1.549**	0.664	0.051
OHIS9	659	1.234	0.247	2.031	0.681	2.637**	1.488	0.153
OHIS10	−409	−0.765	−0.153	0.611	0.205	1.177**	0.767	0.035
OHIS11*	−1,398	−2.618	−0.524	0.308	0.103	--	--	0.011

^†^BWS attributes: OHIS1, information source from trustworthy and authoritative website; OHIS2, verified by professional institutions or health professionals; OHIS3, recommendations from other users; OHIS4, disclosure of site owner, website disclaimer, and contact information; OHIS5, Writing and language; OHIS6, disclosure of author information; OHIS7, consistency of information; OHIS8, currency of information; OHIS9, Privacy and security guaranteed; OHIS 10, professional interface design; OHIS 11, without advertisement links.

*OHIS 11 was chosen as the reference, as it was the least worrisome outcome, so all parameters would be positive; **p < 0.001.

The mixed logit models estimated the coefficient results of the online health information-seeking attributes. The relative importance of each attribute was estimated with reference to the attribute of “without advertisement links,” which was identified as the least important attribute of the 11 online health information-seeking attributes. As expected, all the remaining 10 coefficients were positive and statistically significant, which indicated that they were preferred to the reference attribute of “without advertisement links.” The maximum likelihood estimations reported that “verified by professional institutions or health professionals” (coefficient = 3.096), “information source from trustworthy and authoritative website” (coefficient = 3.015), and “privacy and security guaranteed” (coefficient = 2.637) were the most important attributes of online health information seeking among the respondents. “Consistency of information,” “currency of information,” and “recommendation from other users” were the next important attributes (Table 3).

The share of preference values of the attributes is also presented in Table 3. The share of preference estimates by the mixed logit model confirmed the results of the relative importance of each attribute. “Verified by professional institutions or health professionals” and “information source from trustworthy and authoritative website” were ranked first and second, respectively (Table 3).

### 3.3 Effects of with and without a medical education background on BWS evaluation

The effects of covariates (receiving medical education and gender) were also analyzed in a random parameter logit model. The rank results of each attribute were consistent with previous models, and all the estimates of each attribute were statistically significant after controlling the covariate of receiving medical education (*p* < 0.001). The detailed results of the effects of the covariate of receiving medical education are presented in Table 4. Specifically, receiving medical education had 0.410 and 0.279 positive effects on “writing and language” and “professional interface design,” respectively. On the other hand, the negative effect on “disclosure of author information” (coeff = −0.307) was also calculated in the random logit model. The effects of covariate gender on BWS attribute preferences were also conducted and are presented in Table 4. Compared with males, female college students had positive significant effects on the preference for “information source and verification,” “recommendation from other users,” “disclosure of website and author information,” and “consistency of information” (Table 4).

**Table 4:** Effects of medical education on BWS preference according to the random logit model.

BWS attributes^†^	Coeff	Standard deviation	*p*-value
Effects of receiving medical education			
OHIS1.medical education	−0.190	0.134	0.156
OHIS2.medical education	−0.216	0.138	0.117
OHIS3.medical education	0.217	0.140	0.121
OHIS4.medical education	−0.181	0.119	0.128
OHIS5.medical education	0.410	0.112	<0.001**
OHIS6.medical education	−0.307	0.114	0.007**
OHIS7.medical education	−0.193	0.126	0.125
OHIS8.medical education	0.150	0.114	0.185
OHIS9.medical education	0.297	0.153	0.052
OHIS10.medical education	0.279	0.112	0.012**
OHIS11.medical education*	--		
Effects of gender			
OHIS1.gender	0.508	0.154	<0.001**
OHIS2.gender	0.743	0.162	<0.001**
OHIS3.gender	0.328	0.149	0.040**
OHIS4.gender	0.383	0.139	0.006**
OHIS5. gender	−0.213	0.137	0.119
OHIS6. gender	0.398	0.134	0.003**
OHIS7. gender	0.412	0.147	0.004**
OHIS8. gender	−0.152	0.137	0.267
OHIS9. gender	0.334	0.170	0.050
OHIS10. gender	−0.217	0.130	0.095
OHIS11. gender*	--		

^†^OHIS1, information source from trustworthy and authoritative website; OHIS2, verified by professional institutions or health professionals; OHIS3, recommendations from other users; OHIS4, disclosure of site owner, website disclaimer and contact information; OHIS5, writing and language; OHIS6, disclosure of author information; OHIS7, consistency of information; OHIS8, currency of information; OHIS9, privacy and security guaranteed; OHIS 10, professional interface design; OHIS 11, without advertisement links.

*OHIS 11 was set as a reference level. Bold values indicates **<0.05.

## 4 Discussion

### 4.1 Main findings

In this study, over 90% of participants reported they had searched the Internet for obtaining health information. The high prevalence of online health information-seeking behavior has been observed in Chinese college students and in other study settings (3). Our study corroborates the findings from a demographically comparable cohort of college students, confirming the predominance of “Baidu” as the primary search engine and “healthy lifestyles or behavior” as the most frequent search topic (11). Furthermore, this pattern of search topics is consistent with observations from studies of other Chinese populations and settings (31, 32).

It is also worthy to note that this study examined online health information-seeking behavior during the COVID-19 pandemic. In contrast, the aforementioned study of Chinese college students was conducted in 2018, prior to the outbreak. The previous research has illustrated that the frequency of online health information behavior was increased and was affected by COVID-19 (33, 34). On the other hand, the effects of social media, such as WeChat, TikTok, and Weibo, on online health information seeking should also be given continuous attention. Young people are increasingly receiving health information on social media platforms through both active participation and passive exposure, especially in areas such as physical activity, diet/nutrition, and body image (35, 36).

On the other hand, by using the BWS method, we measured the relative importance of multiple online health information seeking indicator choices among college students. The findings showed that “verified by professional institutions or health professionals,” “information source from trustworthy and authoritative website,” and “privacy and security guaranteed” were the most preferred indicators of online health information seeking among college students, and “without advertisement links” was the least preferred indicator by the respondents.

The two attributes “verified by professional institutions or health professionals” and “information source from trustworthy and authoritative website” were ranked as the first and second most important attributes. The two attributes together summed up to 46.5% (mixed logit estimates) of the 11 choice options selected by the respondents in this study, showing the importance of the 2 attributes. The study results were consistent with previous findings that accuracy and information source were chosen as the most valuable criteria influencing consumers’ health information seeking (37–39). For example, in one cross-sectional study among college students evaluating factors influencing online health information, the findings showed that approximately 98% of respondents rated the accuracy of information as “fairly important” and “very important” based on a 3-point Likert scale (37). However, in terms of accuracy and credibility, in a qualitative study of interviewing 101 undergraduate students, over 85% of the users believed the online health information they consulted was unprofessional, highlighting the importance of improving the professionalism of the online health information (18).

This finding reveals the specific vulnerabilities and corresponding coping strategies used by this cohort in their quest for online health information. As digital natives, college students are exposed to an unprecedentedly complex online information ecosystem, yet they often lack sufficient medical knowledge and digital health literacy to critically assess content. Consequently, this pronounced concern about the infodemic drives their heavy reliance on professional institutions and trustworthy websites as efficient “trust agents” (40, 41). This behavioral pattern is well-articulated by the Health Belief Model: college students may perceive a high susceptibility to health threats posed by misinformation and concurrently a high severity of its potential consequences. In response, they have concluded that deferring to authoritative sources offers the greatest perceived benefits for risk mitigation (42). This reliance was likely cemented by their lived experiences of the COVID-19 pandemic, where repeated exposure to the stark contrast between authoritative and misinformation sources further reinforced their preference for institutional certification (43).

Moreover, the findings revealed that privacy and security were of special concern among college students, which was ranked as the third most important attribute influencing online health information seeking. The college students demonstrated their preference for privacy and security using the best-worst scaling in our study, which was consistent with previous research. A recent survey conducted by the Pew Research Center showed that young adults generally are more focused on online privacy compared with their elders (44). Another research conducted with a focus group with participants aged 19–35 suggested that young adults understand and care about the potential risks associated with disclosing information online and would engage in privacy-protective behaviors on social media (45). Young adults themselves had an awareness of online safety and deployed a variety of safety and security measures, such as using pseudonyms and switching between multiple accounts during online activities (45, 46).

The attributes “without commercial links,” “professional interface design,” and “disclosure of information” were rated as the least important attributes in this study. The respondents valued more attributes related to information source and accuracy than attributes related to website interface design and commercial links. The factors of site links and website appearance were also rated as less important factors in previous studies (38).

On the other hand, in this sample, comparisons of preferences for 11 attributes were also estimated between medical college students and non-medical college students. Compared with non-medical college students, college students receiving medical education had more positive preferences for the attributes “writing and language” and “professional interface design.” Previous studies have tried to explore health information-seeking behavior among medical students. The findings demonstrated that medical students were capable of finding trustworthy health-related information online independent of the search engine used (47). It was likely that medical students themselves already had the ability to search for accurate information and reliable information sources. That would help explain, at least in part, that the medical education background itself had no significant effects on the attributes of accuracy and credibility of health information, as students already acquired the required skills and abilities for credible and accurate health information. Rather, the medical students preferred more of the professional interface design and expression of writing and language. This finding would help provide guidance for developing strategies focused on the professional interface design and expression of online health information for medical students.

There were also some limitations to this study. First, as the respondents were asked to choose the best and worst among the given lists of attributes included in the task, the results are limited to the attributes included in the BWS questions. In addition, selection bias is often a concern, as the samples were randomly selected. Studies with a more representative sample population can be conducted in future studies (48).

### 4.2 Theoretical and practical implications

In this study, BWS provided a simple and transparent way to assess the relative importance of factors influencing online health information seeking. The method guides investigators to priorities that can guide the allocation of resources by indicating which should be done as priorities among a list of possible factors for online health information-seeking behavior (26). The rankings of relative importance of the attributes could help reduce information overload and speed up making decisions. Applying the BWS, our findings showed that accuracy, credibility, security, and privacy of health information were the three most important aspects influencing participants’ online health information behavior among college students.

With the relative importance of ranking results by the BWS method, there is a need to recommend strategies for promoting and facilitating accuracy and credibility of online health information to ensure that consumers can have access to health information with verification and a credible source (49). Measures such as developing tools to measure the accuracy and credibility of health information, and information surveillance can be developed and recommended (50). On the other hand, considering the effects of medical education on the respondents’ preference for online health information seeking, the focus of reforms and policies can be transferred to the professional interface design and expression of writing and language to help utilization for medical students.

## 5 Conclusion

Given our results that the majority of respondents searched the Internet for health information, and “self-care,” “disease prevention,” and “mental health” have become the most frequently searched contents of online health information, more attention should be given to online health information concerning these crucial aspects for college students. Especially, guidance from research or policies should pay attention to young people’s engagement with health-related social media. “Verified by professional institutions or health professionals,” “information source from trustworthy and authoritative website,” and “privacy and security guaranteed” were the three most preferred factors of online health information seeking among college students. The application of the BWS method helps to identify the most important factors, reduce information overload, and provide insights for intervention targeting on the accuracy, credibility, privacy, and consistency of online health information management.

## Data Availability

The datasets presented in this study can be found in online repositories. The names of the repository/repositories and accession number(s) can be found in the article/Supplementary material.
