# Supplementary material for: Understanding the preference of online health information seeking among college students using the best-worst scaling method
Source: Front Public Health. 2025 Nov 5;13:1670106. doi: 10.3389/fpubh.2025.1670106 (PMC12626865; doi:10.3389/fpubh.2025.1670106)
Supplement: Supplementary file 1 [file Supplementary_file_1.docx]

**Supplementary File 1**

**
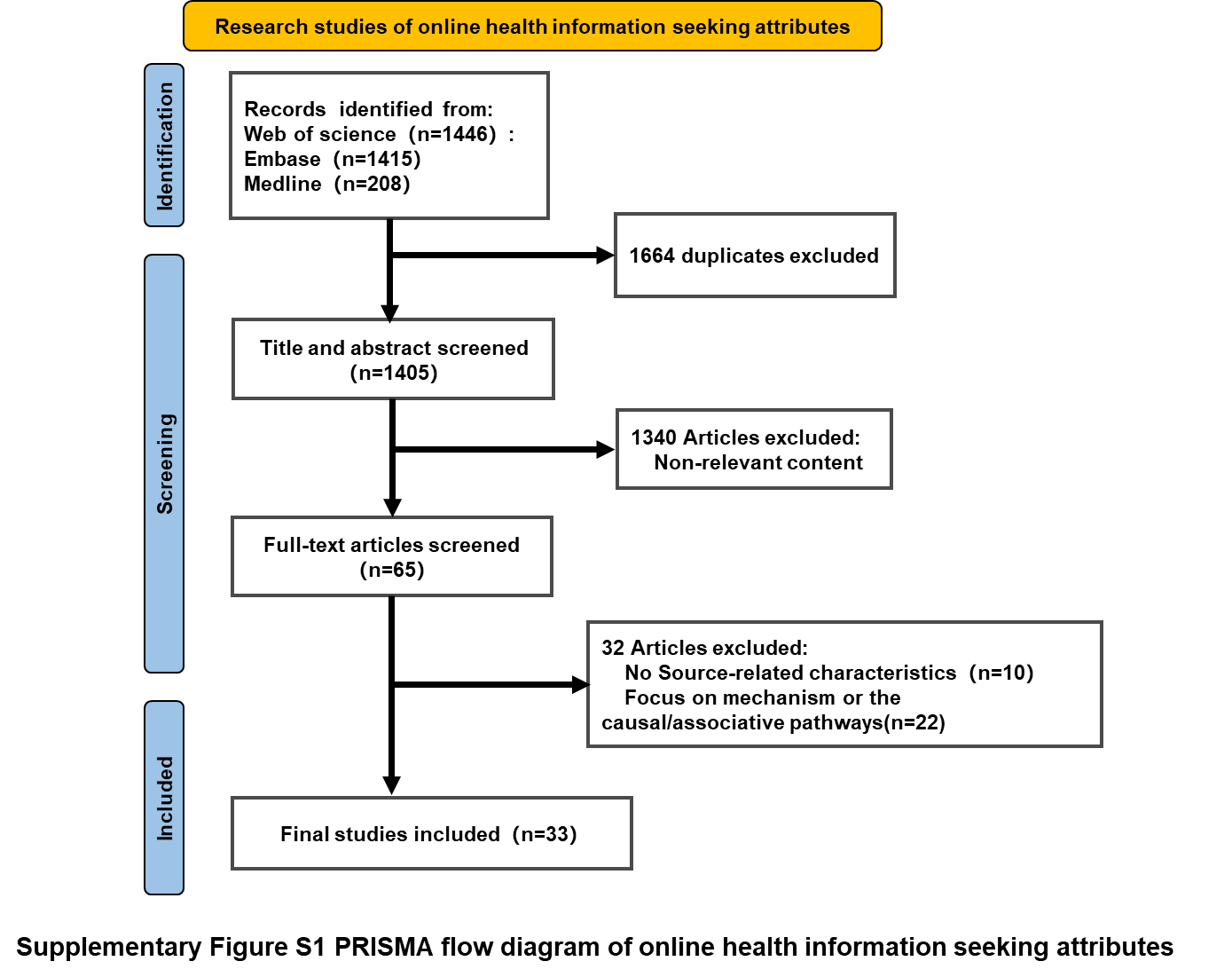
**

| **Supplementary File 2**  **Table S1 Online health information seeking attributes** | | |
| --- | --- | --- |
|  | **Attributes** | **References** |
| 1 | Information source from trustworthy and authoritative website | (Alkan, Küçükoglu, & Ünver, 2024; Ferraris et al., 2023; Mackert M, 2009; Maon S.N., 2017; Osei Asibey B, 2017; Peterson G, 2003; R., 2015; Williams P, 2003; Zhao, Zhao, & Song, 2022) |
| 2 | Verified by professional institutions or health professionals | (Ferraris et al., 2023; Maon S.N., 2017; Osei Asibey B, 2017; Sadah SA, 2015; Zhao et al., 2022) |
| 3 | Recommendations from other users | (Cusack L, 2017; Diviani N, 2016; Feufel MA, 2012; Subramaniam M, 2015) |
| 4 | Disclosure of site owner, website disclaimer and contact information | (C, 2010; Champlin S, 2017; Cusack L, 2017; Freeman KS, 2009; Peterson G, 2003; Sillence E, 2007 ) |
| 5 | Content with factual information, e.g. statistics and numbers | (C, 2010; Cunningham A, 2016 ; Cusack L, 2017; Kerr C, 2006; Scantlebury A, 2017; Shi et al., 2025; Sillence E, 2007 ) |
| 6 | Content linked to references, e.g. original documents | (Crystal A, 2006 ; Frisby G, 2002 ; Lederman R, 2014 ; Payton FC, 2014; Scantlebury A, 2017) |
| 7 | Writing and language | (Cusack L, 2017; Freeman KS, 2009; Lederman R, 2014 ; Maon S.N., 2017; Osei Asibey B, 2017; Payton FC, 2014; Rennis L, 2015; Scantlebury A, 2017) |
| 8 | Disclosure of author information | (Champlin S, 2017; Cusack L, 2017; Diviani N, 2016; Freeman KS, 2009) |
| 9 | Consistency of information | (Alsem MW, 2017; Champlin S, 2017; Cusack L, 2017; Maon S.N., 2017; Osei Asibey B, 2017; Zhao et al., 2022) |
| 10 | Currency of information | (Alkan et al., 2024; Cusack L, 2017; Diviani N, 2016; Maon S.N., 2017; Osei Asibey B, 2017; Rennis L, 2015; Scantlebury A, 2017; Shi et al., 2025) |
| 11 | Without advertisement links | (Champlin S, 2017; Cunningham A, 2016 ; Cusack L, 2017; Diviani N, 2016; Peddie KA, 2017) |
| 12 | Quality of links | (Osei Asibey B, 2017; Sillence E, 2007 ) |
| 13 | Privacy and security guaranteed | (Osei Asibey B, 2017; Peterson G, 2003; Shi et al., 2025) |
| 14 | Overall appearance | (Ben-Mussa A, 2018; Cunningham A, 2016 ; Osei Asibey B, 2017; R., 2015) |
| 15 | Professional interface design | (Cunningham A, 2016 ; Osei Asibey B, 2017; Rafiq, Ya, Wu, & Waqas, 2025; Sillence E, 2007 ; Subramaniam M, 2015) |
| 16 | Security setting | (McPherson AC, 2014; Zhang, 2021) |

**References**

Alkan, O., Küçükoglu, U., & Ünver, S. (2024). Comparison of factors affecting Turkish citizens' search for online health information before and during the COVID-19 pandemic. *BMC PUBLIC HEALTH, 24*(1). doi:10.1186/s12889-024-19546-y

Alsem MW, A. F., Verhoef M, Jongmans MJ, Meily-Visser JMA, Ketelaar M. (2017). Information seeking by parents of children with physical disabilities: An exploratory qualitative study. *Res Dev Disabil, 60*, 125-134.

Ben-Mussa A, P. A. (2018). Popular apps on the medical category targeting patients and the general public in the United Kingdom: do they conform to the health on the net foundation principles? *Health Inform J, 24*, 259-276.

C, M. (2010). How Women With Mental Health Conditions Evaluate the Quality of Information on Mental Health Web sites:A Qualitative Approach. *Journal of Hospital Librarianship, 10*(3), 235-250.

Champlin S, M. M., Glowacki EM, Donovan EE. (2017). Toward a Better Understanding of Patient Health Literacy: A Focus on the Skills Patients Need to Find Health Information. *Qual Health Res, 27*(8), 1160-1176.

Crystal A, G. J. (2006). Relevance criteria identified by health information users during Web searches. *J Am Soc Inf Sci, 57*(10), 1368-1382.

Cunningham A, J. F. (2016). Exploring trust in online health information: a study of user experiences of patients.co.uk. *Health Info Libr J, 33*(4), 323-328.

Cusack L, D. L., Del Mar CB, Hoffmann TC. (2017). A qualitative study exploring high school students' understanding of, and attitudes towards, health information and claims. *Health Expect, 20*(5), 1163-1171.

Diviani N, v. d. P. B., Meppelink CS, van Weert JC. (2016). Exploring the role of health literacy in the evaluation of online health information: Insights from a mixed-methods study. *Patient Education and Counseling, 99*(6), 1017-1025.

Ferraris, G., Monzani, D., Coppini, V., Conti, L., Pizzoli, S. F. M., Grasso, R., & Pravettoni, G. (2023). Barriers to and facilitators of online health information-seeking behaviours among cancer patients: A systematic review. *DIGITAL HEALTH, 9*. doi:10.1177/20552076231210663

Feufel MA, S. S. (2012). What do web-use skill differences imply for online health information searches? . *J Med Internet Res, 14*(3), e87.

Freeman KS, S. J. (2009). Effect of Contact Information on the Credibility of Online Health Information. *IEEE Trans Profess Commun, 52*(2), 152-166.

Frisby G, B. T., Borland R, Anderson JN. (2002). Smoking cessation and the Internet: a qualitative method examining online consumer behavior. *J Med Internet Res, 4*(2), e8.

Kerr C, M. E., Stevenson F, Gore C, Nazareth I. (2006). Internet interventions for long-term conditions: patient and caregiver quality criteria. *J Med Internet Res, 8*(3), e13.

Lederman R, F. H., Smith S, Chang S. (2014 ). Who can you trust? Credibility assessment in online health forums. *Health Policy and Technology, 3*(1), 13-25.

Mackert M, K. L., Tyler D, Gustafson J. (2009). Designing e-health interventions for low-health-literate culturally diverse parents: addressing the obesity epidemic. *Telemed J E Health, 15*(7), 672-677.

Maon S.N., H. N. M., Seman S.A.A. (2017). Online health information seeking behavior pattern. *Adv. Sci. Lett, 23*, 10582-10585. doi: doi: 10.1166/asl.2017.10107

McPherson AC, G. M., Stinson J. (2014). Seeing is believing? A mixed-methods study exploring the quality and perceived trustworthiness of online information about chronic conditions aimed at children and young people. *Health Commun, 29*(5), 473-482.

Osei Asibey B, A. S., Boakye Dankwah A. (2017). The Internet use for health information seeking among Ghanaian university students: A cross-sectional study. *International journal of telemedicine and applications, 2017*.

Payton FC, K. L., Kiwanuka-Tondo J. (2014). Online HIV prevention information: How black female college students are seeking and perceiving. *Internet Research, 24*(4), 520-543.

Peddie KA, K.-C. R. (2017). How people with hearing impairment in New Zealand use the Internet to obtain information about their hearing health. *Computers in Human Behavior, 73*, 141-151.

Peterson G, A. P., Williams KA. (2003). How do consumers search for and appraise information on medicines on the Internet? A qualitative study using focus groups. *J Med Internet Res, 5*(4), e33.

R., B. (2015). Harnessing the Web: How E-Health and E-Health Literacy Impact Young Adults' Perceptions of Online Health Information. *Med 2 0, 4*(2), e5.

Rafiq, S., Ya, C., Wu, J., & Waqas, M. (2025). Mapping the online and offline health information seeking behavior among older adults: a systematic review. *ASLIB JOURNAL OF INFORMATION MANAGEMENT*. doi:10.1108/AJIM-08-2024-0702

Rennis L, M. G., Seidel E, Shneyderman Y. (2015). Google it!: Urban community college students? use of the Internet to obtain self-care and personal health information. *Coll Stud J, 49*(3), 414-426.

Sadah SA, S. M., Wiley MT, et al. (2015). A study of the demographics of WEB-based health-related social media users. *J Med Internet Res*.

Scantlebury A, B. A., Hanley B. (2017). Experiences, practices and barriers to accessing health information: A qualitative study. *Int J Med Inform, 103*, 103-108.

Shi, G., Yu, J. J., Zhang, J. M., Zhao, J., Peng, Z., & Shang, L. (2025). Factors affecting online health information-seeking behavior in young and middle-aged patients with stroke. *PLOS ONE, 20*(4). doi:10.1371/journal.pone.0321791

Sillence E, B. P., Harris P, Fishwick L. (2007). Health Websites that people can trust – the case of hypertension. *Interacting with Computers, 19*(1), 32-42.

Subramaniam M, S. J. B., Taylor NG, et al. (2015). Bit by bit: using design-based research to improve the health literacy of adolescents. *JMIR Res Protoc, 4*(2), e62.

Williams P, N. D., Huntington P. (2003). Health information on the Internet: a qualitative study of NHS Direct Online users. *Aslib Proceedings, 55*((5/6)), 304-312.

Zhang, D., Zhan, W., Zheng, C. et al. (2021). Online health information-seeking behaviors and skills of Chinese college students. *BMC Public Health, 21*, 736.

Zhao, Y. C., Zhao, M. Y., & Song, S. J. (2022). Online Health Information Seeking Behaviors Among Older Adults: Systematic Scoping Review. *JOURNAL OF MEDICAL INTERNET RESEARCH, 24*(2). doi:10.2196/34790
